# HIF-1, Metabolism, and Diabetes in the Embryonic and Adult Heart

**DOI:** 10.3389/fendo.2018.00460

**Published:** 2018-08-15

**Authors:** Radka Cerychova, Gabriela Pavlinkova

**Affiliations:** ^1^Laboratory of Molecular Pathogenetics, Institute of Biotechnology of the Czech Academy of Sciences, Prague, Czechia; ^2^Faculty of Science, Charles University, Prague, Czechia

**Keywords:** hypoxia-inducible factor 1, embryopathy, cardiomyopathy, heart development, fetal programing

## Abstract

The heart is able to metabolize any substrate, depending on its availability, to satisfy its energy requirements. Under normal physiological conditions, about 95% of ATP is produced by oxidative phosphorylation and the rest by glycolysis. Cardiac metabolism undergoes reprograming in response to a variety of physiological and pathophysiological conditions. Hypoxia-inducible factor 1 (HIF-1) mediates the metabolic adaptation to hypoxia and ischemia, including the transition from oxidative to glycolytic metabolism. During embryonic development, HIF-1 protects the embryo from intrauterine hypoxia, its deletion as well as its forced expression are embryonically lethal. A decrease in HIF-1 activity is crucial during perinatal remodeling when the heart switches from anaerobic to aerobic metabolism. In the adult heart, HIF-1 protects against hypoxia, although its deletion in cardiomyocytes affects heart function even under normoxic conditions. Diabetes impairs HIF-1 activation and thus, compromises HIF-1 mediated responses under oxygen-limited conditions. Compromised HIF-1 signaling may contribute to the teratogenicity of maternal diabetes and diabetic cardiomyopathy in adults. In this review, we discuss the function of HIF-1 in the heart throughout development into adulthood, as well as the deregulation of HIF-1 signaling in diabetes and its effects on the embryonic and adult heart.

## Introduction

**Figure 1:**
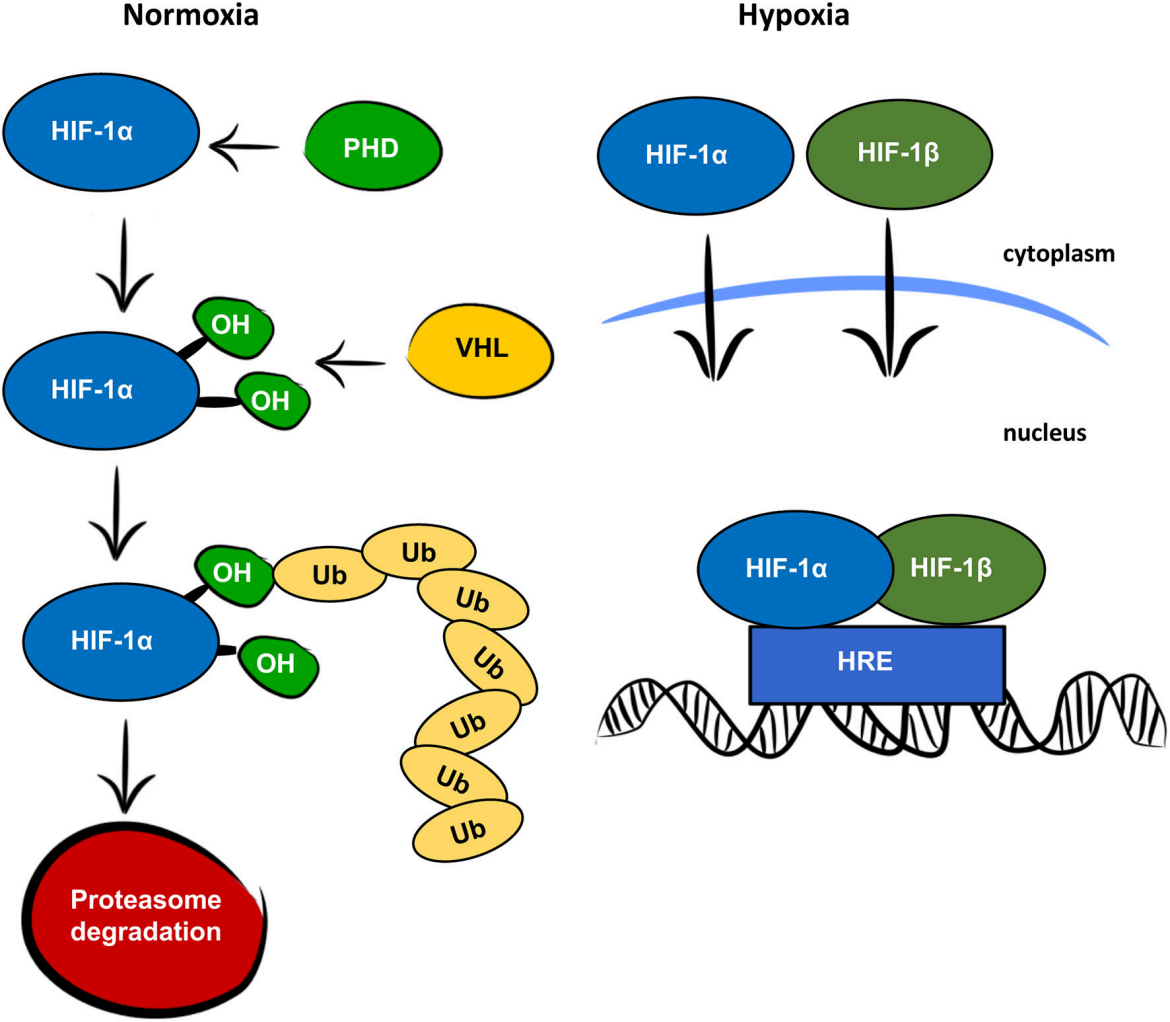
Oxygen dependent regulation of HIF-1α. In normoxic conditions, the HIF-1α protein is recognized by prolyl hydroxylase proteins (PHD), which hydroxylate prolines (OH) in the oxygen-dependent degradation domain. The hydroxylated prolines are recognized by the von Hippel-Lindau protein (VHL) and ubiquitinated (Ub) for degradation by the proteasome. In hypoxic conditions, HIF-1α and HIF-1β are translocated to the nucleus, where they form a heterodimer, bind to the hypoxia responsive element (HRE) and function as a transcription factor.

Oxygen is a key factor in mammalian energy metabolism (1). In normoxic conditions, cells generate adenosine triphosphate (ATP) through the mitochondrial electron transport chain, which requires oxygen as a terminal electron acceptor. In low oxygen concentrations, cells adjust their oxygen consumption and switch to anaerobic glycolysis to prevent oxygen depletion. Glycolysis is a less efficient process of generating ATP but is less oxygen demanding and thus protects cells from total anoxia and cell death. Hypoxia-inducible factor 1 (HIF-1) is the main regulator of responses to hypoxia. It consists of two subunits, HIF-1α and HIF-1β, encoded by the *Hif1a* and *Hif1b* genes, respectively. Both the HIF-1α and HIF-1β subunits are expressed constitutively, but only HIF-1α is affected by oxygen levels, as is shown in Figure 1 (2). Under normoxia, HIF-1α is rapidly degraded. First, prolyl hydroxylase domain proteins (PHDs) hydroxylate prolines in the oxygen—dependent degradation domain. The hydroxylated prolines are then recognized by the von Hippel-Lindau (VHL) protein, which serves as an E3 ubiquitin ligase. Finally, polyubiquitylated HIF-1α is degraded in the 26S proteasome. In hypoxic conditions, HIF-1α is not hydroxylated and both subunits, HIF-1α, and HIF-1β, are translocated to the nucleus, where they form a heterodimer, bind to hypoxia-responsive elements (HREs) on DNA, and activate transcription of hypoxia-responsive genes. As the main regulator of responses to hypoxia, HIF-1 signaling directly or indirectly targets several hundred genes (3). It is responsible not only for the switch from oxidative phosphorylation to glycolysis, but also for other adaptive processes, such as angiogenesis, erythropoiesis, and cell survival. In addition to responses to physiological hypoxia during development and growth, and pathological hypoxia in adult life, HIF-1 also plays an important role in aerobic glycolysis (4). In aerobic glycolysis, HIF-1 upregulates genes related to glycolytic energy metabolism in normoxia, the so-called Warburg effect. The Warburg effect is most often mentioned in relation to cancer cell growth (5), but some studies suggest the importance of aerobic glycolysis in normal proliferating cells as a mechanism for minimizing oxidative stress (6).

In this review, we will discuss the importance of HIF-1 in cardiac development, perinatal remodeling of cardiac metabolism, and in heart function in adults. We will also summarize the role of HIF-1 signaling in the responses to hypoxia in heart development and aberrant HIF-1 regulation in diabetic conditions.

## Hypoxia and HIF-1 signaling during embryonic development

During early development, the embryo is exposed to hypoxia, as was demonstrated by the measurement of human placental and intrauterine levels of oxygen (7, 8). During the first stages of development, the embryo uses pyruvate as the main energy substrate and glycolysis is undetectable (9). When the blastocyst stage is reached, oxygen consumption rapidly increases along with glycolysis, while the consumption of pyruvate decreases in subsequent stages. The switch to glycolytic metabolism leads to the restoration of oxygen consumption to normal levels. Physiologic hypoxic regions occur in normally developing embryos, mainly in the developing neural tube, heart, and intersomitic mesenchyme (10). There are a number of oxygen-sensing pathways, such as the energy and nutrient sensor mTOR, and the nuclear factor (NF)-κB transcriptional response, but the HIF-1 transcription system is a key feature in the cellular response to a low-oxygen environment during embryonic development (11). Although much of mammalian embryogenesis occurs at low oxygen concentrations (≤2%), it is important to note that the ability of the HIF-1 system to response enhanced and spatially extended hypoxia (nonphysiological hypoxia) is limited. Exposure to nonphysiological hypoxia (induced by environmental insult, cardiovascular defects or placental insufficiency) during early developmental stages leads to developmental defects affecting all organ systems (11). Besides intrauterine growth restriction syndrome and low birth weight, the developing heart is the most susceptible to hypoxia-induced defects.

**Figure 2:**
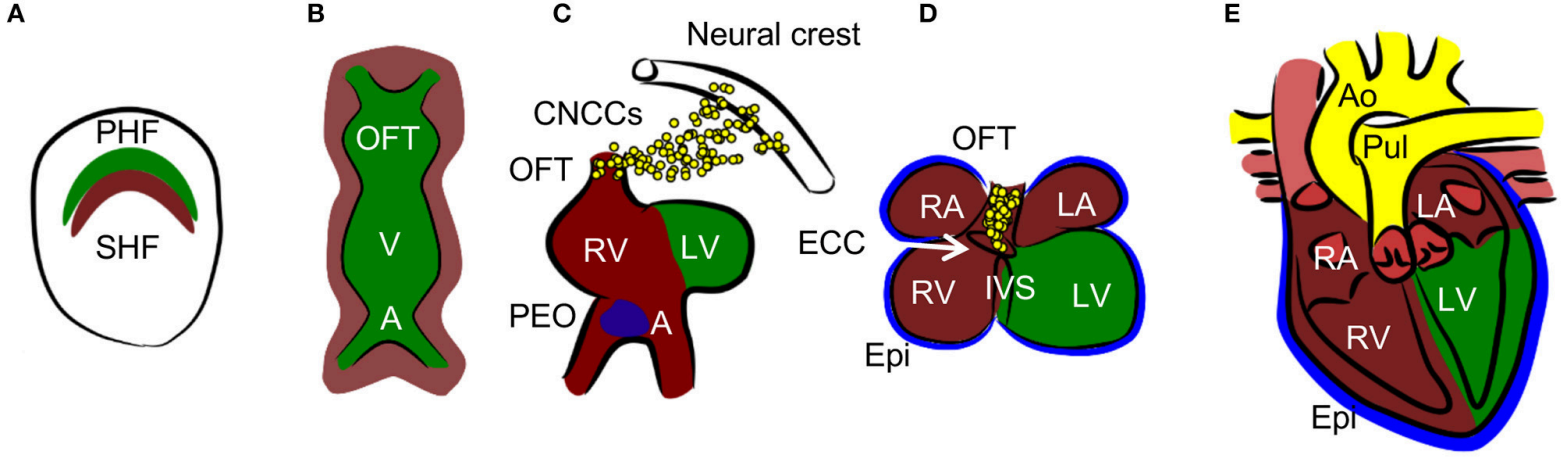
Heart development. **(A)** Cardiac progenitors form crescent shaped primary and secondary heart fields (PHF and SHF). **(B)**The primary heart field then forms the primary heart tube. The future outflow tract (OFT) is in the anterior part, future atria **(A)** are in the posterior part, and future ventricles (V) are in the middle of the tube. Cells from SHF populate the anterior and posterior parts of the tube. **(C, D)** The tube undergoes a process of looping, during which ventricles and atria are formed. The right ventricle (RV), left and right atria (LA and RA), and OFT are formed by SHF cells, the left ventricle (LV) is formed by PHF cells. During the process of septation of the ventricles, the muscular part of the interventricular septum (IVS) grows from the bases of the ventricles and connects with the remodeled endocardial cushion (ECC) in the atrioventricular canal. Similarly, the interatrial septum grows from the roof of the atria. Cardiac neural crest cells (CNCCs), migrating from the neural tube, contribute to the remodeling of the OFT. The proepicardial organ (PEO) is first formed on the ventral side of the future atria and through the process of looping is later positioned dorsally to the heart. The cells from the PEO migrate to the heart tube and form the epicardium (Epi). **(E)** The OFT is septated into the aorta (Ao) and pulmonary truncus (Pul) in the final step of heart development.

Normal cardiac development resulting in the formation of the mature four-chambered heart involves an intricate combination of specifically timed cell migration, proliferation, and differentiation (12) (summarized in Figure 2). The embryo is especially vulnerable to death caused by hypoxia exposure at the time of septation (embryonic day E12.5–E14.5 in mice) (13), which leads to decreased proliferation, resulting in a hypoplastic myocardium. Another study establishes the critical developmental window for heart defects as E10.5–E13.5 (14). At this time point, oxygen deficiency is associated, along with myocardial thinning, with septal defects of ventricles and atria, as well as with defects in the formation of the outflow tract. Experiments with chicken embryos show that increased hypoxia in specific regions of the developing heart is associated with HIF-1α nuclear localization (15, 16). In the outflow tract, hypoxia, and thus HIF-1 signaling, is critical for normal outflow tract remodeling into the great vessels. Nuclear localization of HIF-1α occurs in the interventricular septum (IVS), atrioventricular canal, and the myocardium of the atrial wall at stage 30 when septation is complete. Hypoxic regions follow a pattern which resembles the locations of future coronary vessels, suggesting the importance of HIF-1 signaling in vascularization (17).

Several models of global and conditional *Hif1a* deletion demonstrate the importance of HIF-1 signaling in cardiac development and in embryonic development overall. Global knock-out of the *Hif1a* gene (*Hif1a*^−/−^) was achieved by the replacement of exon 2, encoding the bHLH domain, with a neomycin resistance cassette (18–20). This *Hif1a* mutation is embryonic lethal due to severe cardiovascular defects by E10.5 (18–20). Heart defects of *Hif1a*^−/−^ embryos range from the improper formation of the heart tube, resulting in cardia bifida, to the defective looping of the heart tube, leading to the abnormal formation of the cardiac chambers and defective vascularization (20). Another study shows hyperplastic myocardium in *Hif1a*^−/−^ mutant embryos (18). Additionally, *Hif1a*^−/−^ embryos display neural tube and head fold defects, probably due to increased cell death and defective cell migration from the neural tube. *Hif1a*^+/−^ embryos develop normally. Similarly, embryos with cardiac myocyte-specific deletion *Mlc2v-Cre/Hif1a*^*flox*/*flox*^ develop normally (21). However, the combination of the null allele with *Mlc2v-Cre* (*Mlc2v-Cre/Hif1a*^−/*flox*^*)* results, similarly to the full knock-out model, in heart looping defects, a hyperplastic myocardium, and embryonic death by E12 (22). Increased mRNA levels of genes encoding proteins involved in cell cycle progression and decreased expression of cell cycle inhibitors suggest a role of HIF-1 in the regulation of proliferation. In contrast, another study used a combination of the null allele and *Nkx2.5-Cre* driven deletion of *Hif1a (Nkx2.5-Cre/ Hif1a*^−/*flox*^), which resulted in only 73% of the mutants having a complete deletion, while the remaining mutants had diminished expression of HIF-1α (23). *Nkx2.5-Cre/ Hif1a*^−/*flox*^ mutants with efficient depletion of HIF-1α died by E17.5. Mutants displayed IVS defects and a hypoplastic myocardium due to decreased proliferation of fetal cardiomyocytes, which is in contrast with the study using the *Mlc2v-Cre/Hif1a*^−/*flox*^ model (22) or global deletion of *Hif1a* (18). These contrasting results might be attributed to the different null *Hif1a* allele. Mouse models with the hyperplastic phenotype used *Hif1a*^*tm*1*Jhu*^, affecting all cells derived from the zygote, whereas the model with the hypoplastic phenotype used *Meox2-Cre* for creating the null allele, which is expressed in the epiblast and does not affect the extraembryonic tissue (24). Another example of a mouse model with disrupted HIF-1 signaling resulting in a hypoplastic myocardium is a model utilizing a dominant-negative mutation (HIFdn) that specifically inhibits transcriptional responses mediated by both HIF-1 and HIF-2 in endothelial cells (25). The HIFdn mutation is embryonically lethal at E11.5; mutant embryos display hearts with a thinned myocardium and defective trabeculation. These results suggest the importance of HIF-1 signaling between embryonic and extraembryonic tissues in heart development.

Increased HIF-1α stabilization resulting from the deletion of the *VHL* gene is embryonically lethal at E10.5 to E12.5 due to defects in placental development (26). Even the delayed deletion of *VHL* using tamoxifen-induction at E10.5 results in embryonic lethality (27). Although timed tamoxifen induced deletion of *VHL* at E10.5 was effective in about 25% of the alleles, mutant embryos died between E14.5 and E15.5, possibly because of the impaired vasculature in the yolk sac. Affected embryos also displayed extensive hemorrhaging, liver necrosis, and dilated blood vessels. All these experiments show the importance of HIF-1 signaling during development, and that both upregulation and downregulation are fatal. The role of metabolic changes induced by the deregulation of HIF-1 signaling remains to be elucidated. For now, we can only speculate whether changes in proliferation induced by the deletion of HIF-1α are related to metabolism. However, experiments with cells lacking *Hif1a* show a deregulated glycolytic pathway and decreased proliferation even under normoxic conditions (18).

Our understanding of the roles of the HIF-1 system and hypoxia in embryonic development has improved. However, we still need to fully define the molecular interactions of HIF-1 in embryo morphogenesis. Examples of crosstalk between HIF-1 regulated pathways and other key regulatory pathways reveal additional molecular mechanisms through which HIF-1α and hypoxia can impact embryonic development. For example, HIF-1α interacts with β-catenin and thus connects hypoxia-induced responses to Wnt signaling (28). Another major pathway in development, Notch signaling, is positively modulated in hypoxic regions in embryos (29, 30). HIF-1α binds to the activated intracellular domain of the Notch receptor to activate transcription of target genes such as transcription repressors Hes1 and Hey2 (30). MicroRNAs (miRNAs) represent an additional regulatory mechanism in which hypoxia-HIF-1α action contributes to development. miRNAs are 21–23 nucleotide non-coding RNAs that regulate gene expression at the post-transcription level and play important roles in many biological processes and pathologies, including development and cardiovascular diseases (31). Of the known hypoxia-responsive miRNAs, miR-210 is the most consistent miRNA regulated by HIF-1α and upregulated during heart development (32–34). Another hypoxia-regulated miRNA detected early during heart development is miR-133 that regulates a cardiac-enriched transcription factor, serum response factor, responsible for the regulation of sarcomeres in the heart (35). Thus the hypoxia-HIF-1α-dependent miRNA-mediated response represents epigenetic regulation in the developing heart.

Highly proliferative cancer cells use aerobic glycolysis even in well-perfused tumors (5, 36). Although this approach is less energetically efficient, Vander Heiden et al. (36) hypothesize that proliferating cells need not only ATP but also carbohydrates as a building material. Using aerobic glycolysis allows the redirecting of some carbohydrates to the production of essential cellular building blocks, such as nucleotides and amino acids. A recent metabolomics analysis of developing embryos shows such redirection of glucose-derived carbons into the pentose phosphate pathway (37). Interestingly, the changes in cellular metabolism are not abrupt, but rather follow the anteroposterior gradient along the neural tube, which suggests an involvement of factors other than just the level of oxygen. Clearly, more research is needed in this field.

## Perinatal remodeling

After birth, cardiomyocytes undergo a number of changes (summarized in Table 1), resulting in the maturation of cardiac tissue. Most importantly, cardiac metabolism switches from the predominant use of glycolysis to the β-oxidation of lipids. Fetal glycolysis is favored not only due to the hypoxic environment *in utero* but also due to substrate availability. Glucose is the main substrate crossing the placenta to the fetal circuit (38). The fetal heart is adapted to glycolytic metabolism by the expression of specific metabolic gene isoforms, such as hexokinase I, pyruvate kinase 4, fetal phosphofructokinase, and M-lactate dehydrogenase (39, 40). Metabolism and gene expression gradually change after birth until weaning. Some studies show that the shift in the expression of genes involved in fatty acid metabolism begins in the second trimester, as shown in the microarray analysis of human fetal hearts (41). One of the potential regulators of this process is NEPAS (neonatal and embryonic Per-Arnt-Sim basic helix-loop-helix protein), a negative regulator of HIF-mediated gene expression, which is expressed in murine embryos and neonates from E15.5 to postnatal day 15 (P15) (42). The shift in metabolism might be not only time dependent, but also tissue-specific. At mid-gestation, HIF-1α is expressed in the compact myocardium but not in the trabeculae (43), which leads to a metabolic shift from glycolysis in the compact myocardium whereas oxidative metabolism increases in the trabeculae. The maturation of cardiac oxidative metabolism is completed after birth, although the mechanism is still not fully understood. An increased amount of oxygen, resulting in decreased HIF-1 signaling, is thought to be the main impulse for the shift. Additionally, substrate availability, and composition change after birth because breast milk also contains lipids which are not transferred across the placental barrier during prenatal development (40).

**Table 1:** Perinatal remodeling: a list of the main changes in cardiomyocytes during the transition from the prenatal to the postnatal phenotype.

	**Prenatal heart**	**Postnatal heart**
Number of nuclei	1 nucleus	1-2 nuclei
Main source of energy	Glycolysis	Oxidative phosphorylation
Mitochondria	Small, immature	Large, with dense cristae
Myofibrils	Poorly organized β-myosin heavy chain Slow skeletal muscle isoforms	Organized along the long axes α-myosin heavy chain Cardiac muscle isoforms
Adherence junctions	Surround the cells	Organized at the site of myofibril attachment

The major part of ATP production in the adult heart (over 95%) is provided by mitochondrial oxidative phosphorylation (44). The maturation of mitochondria is an important part of perinatal cardiac remodeling. Fetal mitochondria are small and round; however, after birth, mitochondria fuse, grow bigger, and the cristae of the inner membrane become denser and more uniform (45). The total area of mitochondria per cardiomyocyte triples between E16.5 and P10.5. This process is dependent on the downregulation of HIF-1 signaling. Mitochondria in cardiomyocytes of α*MHC-Cre/VHL*^*flox*/*flox*^ mice appear immature and the cells are more dependent on glycolysis than on oxidative phosphorylation. In *wild type* mice, perinatal cardiomyocytes are dependent on glycolysis as well as on oxidative phosphorylation.

Adult cardiomyocytes combine qualities of striated muscles and smooth muscles, so they can produce great force without any need for a pause. Similar to striated muscles, cardiomyocytes contain sarcomeres as contractile units but, unlike skeletal muscle, they do not form a syncytium and instead become binuclear via incomplete division. Cardiac beating is in fact the synchronized shortening of sarcomeres in cardiomyocytes. Synchronization is achieved by the gradual transfer of the signal from one cell to another through intercellular contacts. During embryonic development, cells are surrounded by adherence junctions and the myofibrils are poorly organized (46). At the neonatal stage, the junctions are organized at sites of myofibril attachment, which are gradually organized along the long axis of the cell. The isoform transition of sarcomeric proteins is induced by an increased force-generating capacity and Ca^2+^ sensitivity (47). Slow skeletal muscle isoforms of troponin-T and troponin-I expressed in the fetal heart are replaced with cardiac troponin-T and troponin-I isoforms. The beta-myosin heavy chain (β-MHC) is replaced by an α-MHC and the ratio of total MHC to actin proteins increases after birth. The β-MHC to α-MHC isoform switch is probably driven by a decrease in HIF-1 signaling. An *in vitro* study shows an increased expression of the *Myh7* gene encoding the β-MHC in reaction to hypoxia and/or stabilized HIF-1α (48). α-MHC fibers work faster than β-MHC (49) and require more ATP molecules (50). The switch to oxidative phosphorylation allows more energy to be spent in order to produce a more efficient contraction of the heart.

## HIF-1 signaling and adult heart function

The contraction of cardiomyocytes is driven by a process called excitation-contraction coupling. This means that the depolarization of the sarcolemma triggers a cascade of processes leading to contraction (51). Upon depolarization, voltage-dependent L-type Ca^2+^ channels are activated, so a small amount of Ca^2+^ is quickly released into the cell and activates ryanodine receptors (RyR) in the sarcoplasmic reticulum (SR) responsible for the release of a large amount of Ca^2+^ into the cytosol. Ca^2+^ then interacts with troponin C in the troponin-tropomyosin complex located on thin actin filaments leading to a conformational change, and binding of the globular heads of myosin filaments.

The relaxation of cardiomyocytes is mainly dependent on the active sequestration of Ca^2+^ by SERCA-2, an ATP-dependent Ca^2+^ pump, located in the membrane of SR. Per every two moles of Ca^2+^ transferred into the lumen of the SR, one mole of ATP is used. Under normal conditions, adult heart glycolysis represents only a minor part of energy production. The majority of ATP is produced by the oxidative phosphorylation of fatty acids (FA). However, the heart is considered to be a true omnivore, which means that under certain circumstances, lactate, ketone bodies or even acetate can be used as energy substrates (52). Since neonatal remodeling is accompanied by glycogen storage depletion and the heart has a small capacity for storage or *de novo* synthesis of FA, the main source of energy substrates is the blood. The metabolic adaptations to hypoxia exposure are determined by the degree and duration of hypoxia (53). Chronic exposure to moderate hypoxia, as in high-altitude inhabitants, leads to the development of defense mechanisms against acute oxidative stress. Adaptive mechanisms provide increased oxygen delivery through increased erythropoiesis and angiogenesis and metabolic remodeling in favor of carbohydrate utilization.

Acute hypoxia induces anaerobic glycolysis in order to compensate for cellular energy demands and actively suppresses oxidative phosphorylation (54). HIF-1 directly activates pyruvate dehydrogenase kinase 1 (PDK1), which inactivates pyruvate dehydrogenase, which is a part of pyruvate dehydrogenase complex that converts pyruvate into acetyl-CoA. Thus, pyruvate is redirected from the tricarboxylic acid cycle (TCA) and converted into lactate. This leads to the attenuation of mitochondrial respiration and to reactive oxygen species (ROS) production.

The functional role of HIF-1 signaling in the changes induced by hypoxia is shown by experiments with modulated HIF-1α expression. Constitutive cardiac-specific over-expression of *Hif1a* leads to changes in cellular metabolism and increased glucose utilization (55), resulting in cardiomyopathy in aging mice. Reversible cardiac specific introduction of the oxygen-stable form of HIF-1α also leads to changes in the expression of metabolic genes in favor of glycolysis and negatively affects heart function, with changes being detectable as early as 3 days after HIF-1α induction (56). Worsening cardiac function with time results in cardiac hypertrophy without detectable histological pathology. Heart function recovers after the *Hif1a* transgene is turned off, which indicates that transient expression of the oxygen-stable transgene induces only reversible changes. However, the reversible nature of these changes in this study may be explained by the fact that the expression of the *Hif1a* transgene was turned-off after only seven days. Mutants with altered HIF-1 signaling, transgenic mice with an α*MHC-Cre* driven cardiomyocyte-specific deletion of *Phd2* and *Phd3*, and mice with a cardiomyocyte-specific deletion of *Vhl*, show decreased cardiac function and premature mortality (57, 58). Consistent with the effects of HIF-1 on cellular metabolism, hearts lacking PHD2 and PHD3 or lacking VHL accumulate glycogen and lipids. Similarly, cardiac myocyte-specific deletion of HIF-1α negatively affects heart contractility and leads to the decreased expression of genes involved in glucose metabolism (21).

## HIF-1 signaling in diabetes

**Figure 3:**
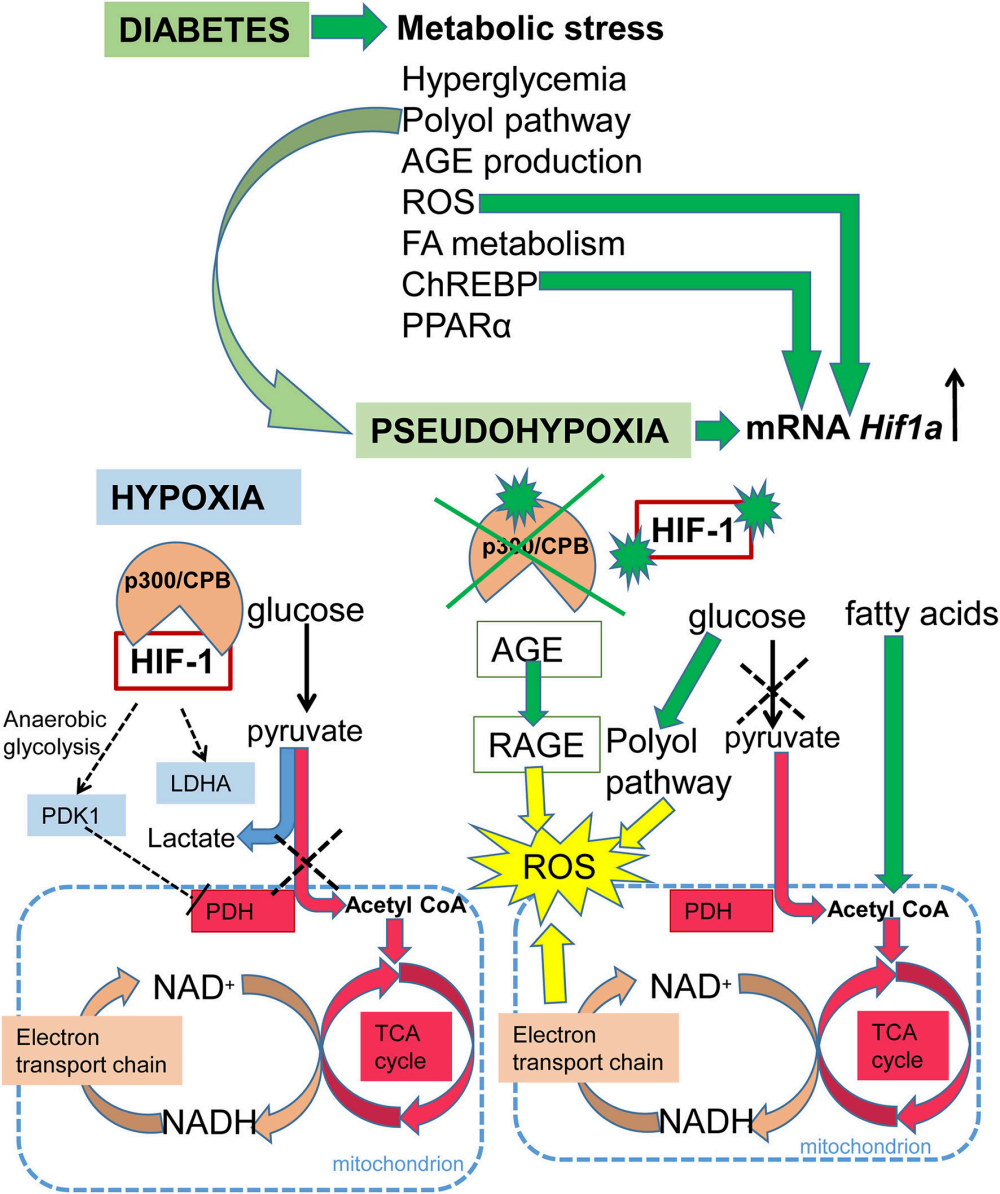
Schematic summary of HIF-1 signaling in hypoxia and diabetes. Under normoxia, glucose is metabolized to pyruvate and then the pyruvate dehydrogenase complex converts pyruvate to acetyl-CoA, which enters the TCA cycle, and NADH and FADH_2_ are generated. Electrons derived from NADH and FADH_2_ are utilized by the electron transport chain to generate ATP. Under hypoxia, HIF-1 activates glycolytic genes as a critical step of metabolic adaptation to hypoxia. HIF-1 induces anaerobic glycolysis by mediating the expression of pyruvate dehydrogenase kinase 1 (PDK1), which inhibits pyruvate dehydrogenase (PDH) and induces conversion of pyruvate to lactate. Under diabetes, metabolic stress is induced by: an increased ratio of NADH/NAD^+^ due to an increased flux of glucose through the polyol pathway resulting in pseudohypoxia; increased expression of peroxisome proliferator-activated receptor alpha (PPARα), which induces fatty acid utilization and oxidation; increased fatty acid oxidation leading to the production of acetyl-CoA; increased production of advanced glycation end products (AGE), which leads to the irreversible modification of proteins; induction of the carbohydrate response element binding protein (ChREBP); and increased ROS production. The production of ROS is induced by oxidative stress due to the increased ratio of NADH/NAD^+^, and by AGE *via* signaling through its receptor (RAGE). ROS, ChREBP, and pseudohypoxia induce transcription of *Hif1a* mRNA. However, in the diabetic environment, HIF-1 signaling is negatively affected *via* modification of its coactivator p300, and by HIF-1α protein modifications.

**Figure 4:**
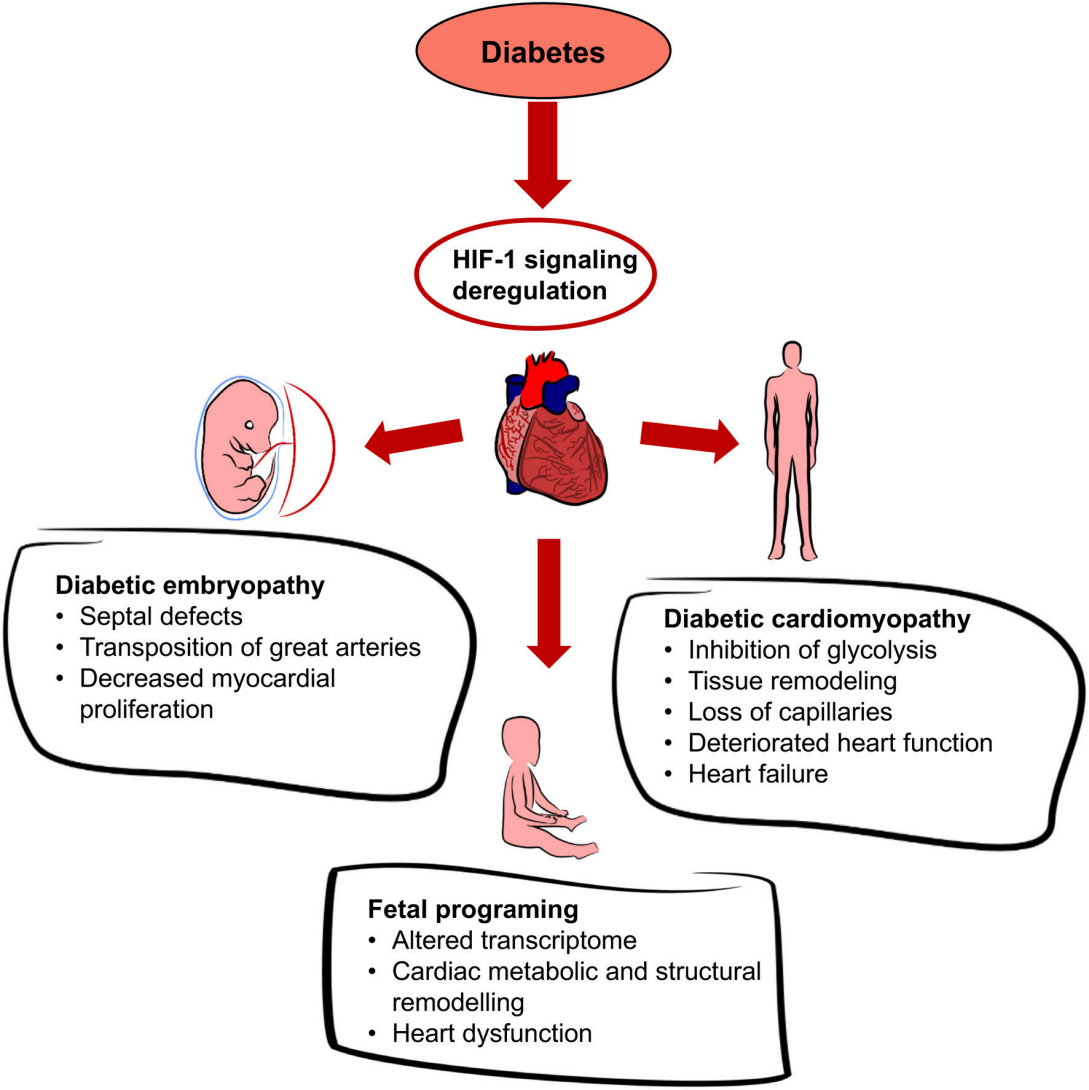
Effects of diabetes on the heart. Diabetes leads to systemic hyperglycemia and hyperglycemia increases tissue hypoxia. HIF-1 signaling is altered due to the changes in HIF-1α transactivation and HIF-1α protein modifications. Dysregulation of cardiac HIF-1 signaling is associated with an increased risk of diabetic embryopathy, adverse long-term outcomes for the offspring, and diabetic cardiomyopathy.

Diabetes is a disease affecting glucose homeostasis regardless of the etiology (59). It affects different organ systems in the body, causing severe complications, such as cardiovascular diseases, diabetic nephropathy, neuropathy, retinopathy, and impaired wound healing. The diabetic environment also negatively affects the developing fetus. Diabetic pregnancies are associated with an increased incidence of congenital malformations (diabetic embryopathy) compared with non-diabetic pregnancies. In addition to the direct teratogenicity of maternal diabetes, the intrauterine and early postnatal environments can influence the cardiovascular and metabolic health of the offspring later in life. Here, we focus on the effect of hyperglycemia on HIF-1 signaling, as summarized in Figure 3. Tissue exposure to hyperglycemia is considered to be the main factor in the development of complications, but even tight regulation of glycemia is not sufficient enough to completely prevent them. Hyperglycemia is often associated with a phenomenon called pseudohypoxia (60), which is characterized by an increased ratio of NADH/NAD^+^ due to an increased flux of glucose through the polyol pathway (61). In hypoxic conditions, the balance is disturbed via impaired oxidation of NADH (60). Some authors disagree with the pseudohypoxia hypothesis, since their results show no changes in the amounts of NADH and NAD^+^ when isolated diabetic and non-diabetic rat retinas are compared (62). Nevertheless, analyses using pimonidazole, a marker for hypoxic tissue, show increased levels of hypoxia in diabetic tissue (63, 64) and the expression of *Hif1a* mRNA is elevated in diabetic rat hearts (65). At the protein level, HIF-1α is stabilized not only by hypoxia but also by products of aerobic glycolysis, mainly pyruvate (4). In contrast, hyperglycemia affects HIF-1 transactivation via modification of its coactivator p300 and thus, diminishes HIF-1 transcriptional activity (66) without affecting the stability of the HIF-1α protein (67). High glucose also activates HIF-1-mediated signal transduction *via* a glucose-responsive carbohydrate response element binding protein (ChREBP), as shown in a model of diabetic glomerulopathy (68). ChREBP plays a functional role in glycolytic and lipogenic gene regulation (69). The next level of diabetes-induced deregulation of HIF-1 signaling involves responses to hypoxia. Experiments with cells cultured in an environment combining hyperglycemia and hypoxia show increased degradation of the HIF-1α protein (70, 71). Thus, diabetes not only causes hypoxia but also compromises HIF-1 signaling. The inability of cells/tissues to appropriately respond to hypoxia increases the risk for complications in diabetic patients. For example, the expression of *Hif1a* mRNA is lower and the size of the affected area is bigger in the infarcted hearts of streptozotocin-induced diabetic rats than in non-diabetic controls (65). In the following section, we discuss the effects of deregulated HIF-1 signaling in diabetes on the developing and adult heart, as summarized in Figure 4.

## Diabetic embryopathy

The main energy metabolic substrate in the embryo is glucose, which is obtained from maternal blood by facilitated diffusion across the placental barrier (72). Therefore, maternal hyperglycemia leads to embryonic hyperglycemia. Maternal diabetes has a teratogenic effect on the development of all organ systems; the most commonly affected are the cardiovascular, nervous, and skeletal systems (73, 74). Out of the cardiovascular defects, ventricular septal defects, and transposition of the great arteries are the most common (75). Although the mechanisms of diabetic embryopathy are not fully understood, the role of hyperglycemia is crucial. Studies using mouse models show that hyperglycemia without systemic diabetes has teratogenic effects (76). Since maternal diabetes-induced defects are similar to the defects induced by hypoxia exposure (77), the deregulation of HIF-1 signaling may play a crucial role. This idea is supported by an experiment with *in vitro* cultivation of embryos, which shows that hyperglycemic conditions increase oxygen depletion (78). Moreover, microarray analysis of maternal diabetes-exposed embryos showed that HIF-1 signaling pathways are significantly affected (79), and embryos with global *Hif1a* insufficiency display increased susceptibility to diabetic embryopathy (63). Diabetic *Hif1a*^+/−^ embryos suffer from increased lethality, and an increased number of neural tube and cardiovascular defects. Additionally, the hearts of diabetic *Hif1a*^+/−^ embryos are severely hypoplasic with an increased trabecular layer. The detected decrease in the proliferation of the compact myocardium suggests that cells in *Hif1a*^+/−^ diabetic hearts have an altered developmental program. This has been further confirmed by mRNA analysis, which shows changes in genes crucial for embryonic heart development, such as *Tbx5, Nkx2.5, Nppa*, and *Mef2c*. Interestingly, *Vegfa* expression was deregulated in the diabetic hearts of both genotypes. *Vegfa* is a known target of HIF-1 signaling (80) and both its downregulation and upregulation is embryonically lethal (81, 82). In this context, downregulation of *Vegfa* in embryonic hearts with severe hyperglycemia (63) is an extremely interesting finding.

The causative role of decreased expression of the HIF-1α protein was shown in diabetic embryonic vasculopathy (83). The disadvantage of this study, in terms of applying it to heart development, lies in its focus on early development before the heart forms. Since it was shown that the kinetics of HIF-1α in response to hypoxia differ between individual organs in adult mice (84), different organs should be studied separately. Another complication for the understanding of pathologies associated with deregulated HIF-1 signaling during embryonic development is represented by rapid changes in gene expression in different developmental stages (85, 86).

Besides diabetic embryopathy, altered HIF-1 signaling may influence the long-term outcomes of the offspring from a diabetic pregnancy. Specifically, it may increase the predisposition of the offspring of diabetic mothers to cardiovascular and metabolic diseases in the process of fetal programing (87). Maternal diabetes leads to accelerated worsening of heart function in the *Hif1a*^+/−^ offspring when compared to littermate controls. Many processes are affected in *Hif1a*^+/−^ offspring hearts, including transcriptional changes associated with apoptosis, blood vessel physiology, inflammatory responses, and metabolism (87). Particularly, changes in the expression of *Cd36*, a HIF-1 target gene encoding a multifunctional receptor mediating the uptake of lipoproteins and lipoprotein-derived fatty acids, indicate changes in myocardial substrate selection and utilization in energy metabolism that play a role in the development of cardiac pathologies (87).

## Diabetic cardiomyopathy

In adults, diabetes is associated with an increased risk of cardiovascular diseases, mainly atherosclerosis (61). However, diabetes causes heart dysfunction independently from coronary artery disease and hypertension, diabetic cardiomyopathy (88, 89). Changes leading to the development of diabetic cardiomyopathy involve structural changes in the myocardium, such as increased fibrosis, lipid accumulation, formation of advanced glycation end products (AGE), and changes in myosin isoforms, as well as changes in substrate utilization, increased apoptosis, and mitochondrial dysfunction. Although, the heart is able to utilize any substrate for the generation of ATP, in diabetic conditions, utilization of glucose decreases, whereas the utilization of fatty acids increases. This is caused by the decreased expression of glucose transporter GLUT1, and glycolytic enzyme hexokinase II. These genes are targets of the HIF-1 pathway (90). In this context, the molecular interactions between HIF-1 and other nuclear partners involved in hypoxia-mediated responses, such as NF-κB, CREB, HMGA1, and HIF-2α, need to be mentioned. For example, HMGA1 is transcription factor regulating chromatin structure and gene expression (91). Defects in HMGA1 expression have been associated with insulin resistance and increased susceptibility to type 2 diabetes (92) and increased risk of acute myocardial infarction (93). HIF-2α, a paralog of HIF-1α, is the critical effector in obesity-associated cardiomyopathy (94, 95). Obesity represents an independent risk factor for cardiovascular disease [reviewed in Poirier et al. (96)] that alters the metabolic profile and induces structural cardiac adaptation by excessive lipid accumulation. Obesity affects the heart through multiple mechanisms such as dyslipidemia, glucose intolerance, hypertension, and inflammation. The inflammatory microenvironment contains small molecules such as ROS, NO, and different cytokines with the ability to activate HIF-1 and HIF-2 signaling [as reviewed in Dehne and Brune (97)]. HIF-1α activation during inflammation results in upregulation of classical target genes like VEGF, GLUT1 or metalloproteinases but also in the activation of unique transcripts such as beta 2 integrin, adenosine receptors or chemokine receptors, indicating that HIF-1 has an immune function (98). Obesity and diabetes are associated with increased levels of FA. Increased usage of FA leads to increased consumption of oxygen, which likely exceeds the FA oxidation capacity and generates tissue hypoxia (99–101). Utilization of FA is regulated by peroxisome proliferator-activated receptor α (PPARα), which targets most of the genes of the fatty acid oxidation pathway (102), is inhibited by HIF-1 (103) and its cardiac overexpression leads to inhibition of glucose uptake, mimicking the effect of diabetes (104). Increased β-oxidation of FA also leads to increased production of ROS and increased expression of mitochondrial uncoupling proteins (UCP) (105), which are thought to decrease generation of ROS (106). UCP2 and UCP3 are expressed in the heart and are targets of PPARα signaling (107, 108) which links their activity to the level of FA. Although uncoupling serves as protection against ROS, it also lowers ATP production and thus affects the efficiency of the heart (105, 109). ROS production is also triggered by AGE via signaling through its receptor (RAGE) (110). Both, ROS and AGE not only modify proteins in extra- and intracellular milieu, but also increase oxidative stress, which activates inflammatory pathways. Even without diabetes, chronic inflammation is a cause of cardiac dysfunction (111). ROS trigger HIF-1 signaling in several ways (112, 113). In general, ROS induce HIF-1α. However, as mentioned earlier, HIF-1α is increasingly degraded in hyperglycemic conditions.

miRNAs are additional important regulatory factors in cardiovascular disease, including diabetic cardiomyopathy (114). Each miRNA regulates from a few up to hundreds of target genes in normal development, growth, and function, as well as in disease. miRNAs play an important role in responses to hypoxia and/or diabetes (115–117). Most miRNAs identified with the hypoxia signature are directly regulated through HIF, as recently reviewed (115). miRNAs also regulate expression or stabilization of HIF-1α by direct binding to its mRNAs or by regulation of HIF complex units such as VHL or PHD (115). Differential expression profiles of the most abundant cardiac miRNAs, miR-1 and miR-133a, are linked to diabetic cardiomyopathy (117). miR-133a is downregulated in the diabetic heart (118), and decreased miR-133 levels induce cardiac hypertrophy (119). Moreover, experiments with isolated cardiomyocytes show increased apoptosis in connection with a decreased expression of miR-133a in hypoxic conditions (120). In line with this finding, apoptotic proteins, caspase −8, −9, and −3 are repressed, while the expression of BCL-2 increases in cells overexpressing miR-133a (120). Overexpression of miR-133a also mitigates cardiac fibrosis (119). One of the metabolic proteins targeted by miR-133 is GLUT4 (121). Furthermore, miR-133a inhibits expression of DNA methyltransferase 1 in cardiomyocytes and thus induces changes in the methylation program and overall induces a much broader effect in gene regulation (122). Downregulation of miR-1 causes hypertrophy and fibrosis in the heart, similar to the effects of miR-133a (123). Hyperglycemia induces apoptosis *via* increased miR-1 in cultured cardiomyocytes that blocks the protective capacity of insulin like growth factor-1 (124). In a rat cardiac model of ischemia/ reperfusion, as well as in cardiomyocytes subjected to hypoxia/reperfusion, inhibition of miR-1 increases BCL-2 expression and thus protects against hypoxia/reoxygenation injury (125). Apoptosis is also regulated by miR-195, which is upregulated in diabetic hearts (126). Its downregulation in diabetic mice decreases myocardial hypertrophy, ROS production, and apoptosis. Analysis of interfibrillar mitochondria isolated from diabetic hearts shows increased expression of miR-141, which inhibits translation of phosphate transporter SLC25A3, causing decreased activity of ATP synthase due to lack of substrate (127). Interestingly, the subsarcolemmal mitochondrial population remains unaffected. Upregulation of miR-210 is associated with cardiac hypertrophy and reactivation of the fetal transcriptional program, triggering pathological changes in the myocardium of the human failing heart (34, 35). miR-210 and miR199a/b were modulated by hypoxia and high glucose in cardiomyocytes and dysregulated in diabetic ischemic heart failure patients (114). miRNAs also represent potential diagnostic markers of diabetic complications, for example levels of circulating miR-1 and miR-133 in the serum of patients with type 2 diabetes correlate with myocardial steatosis, an early manifestation of cardiac pathology (128). The above description is only a narrow representation of miRNAs as regulators of cardiac diseases. These findings not only provide novel insights on the roles of miRNA in cardiac pathologies but also identify potential therapeutic targets for reducing cardiac injury and heart failure.

Diabetes is known to impair microvasculature (129, 130); particularly, diabetic cardiomyopathy is associated with a loss of coronary capillaries that further increases tissue hypoxia (131). Since the combination of hypoxia and hyperglycemia increases degradation of the HIF-1α protein (70, 71), the ability of the diabetic heart to respond to hypoxic conditions is compromised (101). Consistent with these data are experiments with altered HIF-1 signaling in mouse hearts. For example, the cardiac-specific overexpression of *Hif1a* in the streptozotocin-induced diabetic mouse model prevents the decrease in GLUT1 and hexokinase II expression as well as hexokinase activity (132). These proteins are substantial in the first two steps of glycolysis: glucose transport into the cell (133) and its phosphorylation (134). These effects of *Hif1a* overexpression on glycolysis may be one of the reasons for the normalization of the amount of ATP generated in *Hif1a* overexpressing diabetic hearts. Similar results were obtained using insulinopenic mice overexpressing hexokinase (135). Additionally, *Hif1a* overexpression in the diabetic heart preserves VEGF expression and angiogenesis, and prevents myocardial fibrosis and remodeling (132). Experiments using the *Hif1a* heterozygous deficient diabetic mouse model further underscore the importance of proper HIF-1 signaling (136). *Hif1a*^+/−^ mice exposed to only 5 weeks of diabetes develop decreased fractional shortening, undetectable in control mice. Changes are also found at the molecular level, such as a decreased amount of VEGFA, an important target of HIF-1, affecting vascularization, and levels of gap-junctional protein connexin43 (Cx43) phosphorylated at serine 368. The phosphorylation of connexins influences intercellular communication (137) and is thus likely to affect heart function, in which proper signal transduction is crucial for contraction. Diabetic *Hif1a*^+/−^ mice also have increased collagen type I levels, which is associated with fibrosis.

## Conclusion

In this review, we focus on the role of HIF-1 in metabolism in embryonic development and in the adult heart. HIF-1 signaling regulates the cellular switch from oxidative phosphorylation to glycolysis. During embryonic development, glycolysis allows the redirection of glucose-derived carbons into the pentose phosphate pathway and thus creates anabolic building blocks for proliferating cells. Embryonic lethality of the global deletion of *Hif1a* due to associated cardiovascular defects underscores its importance for development. However, our knowledge of embryonic metabolism is still incomplete. A comprehensive molecular and cellular understanding of HIF activity in embryonic development will provide a basis for stem cell research and regenerative medicine. An increased level of oxygen and decreased activity of HIF-1 are key factors in the perinatal remodeling of the myocardium. During this stage, the heart undergoes a gradual transition to aerobic metabolism. In adulthood, the cardiac function of HIF-1 signaling is suppressed, but HIF-1 is still readily available for intervention in case of tissue hypoxia. The current state of knowledge suggests HIF-1 signaling is one of the crucial regulators of developmental changes in cardiac metabolism. In this review, we also addressed the effect of diabetes on HIF-1 signaling. Hyperglycemia causes hypoxia, but also affects the function of HIF-1α. The combination of hyperglycemia and hypoxia decreases the stability of HIF-1α, leading to the deregulation of HIF-1 signaling. Experiments using embryos with *Hif1a* global hemizygous deletion show that the deregulation of HIF-1 signaling contributes to the development of diabetic embryopathy and associated cardiovascular defects. Furthermore, *Hif1a* hemizygous mutant offspring of diabetic mothers have worsened heart function compared to their *wild type* littermates. In diabetic cardiomyopathy, consistent with the decreased function and stability of HIF-1α, the myocardium loses its ability to use glucose as a metabolic substrate. Changes in metabolism are not the only pathology induced by diabetes, but with hyperglycemia being the primary trigger in diabetes, this raises the question of whether deregulated metabolism is the origin of these dysfunctions, or just one of the consequences. Resolving this particular question may be the key to developing an efficient therapy. The greatest limitations of HIF biology research are the experimental tools used and the limitations posed by experimental models. For example, hypoxia/HIF-1 responses vary between cell lines and between models, which may affect overall conclusions. Additionally, regulation of HIF-1 signaling is a very complex process that includes regulation of gene transcription, protein stability, and protein-protein interactions. Responses to the hypoxic environment range from diversified cellular modulations to systemic responses.

Research on hypoxia and HIF-1 regulation has significantly improved our understanding of their role in health and diseases. However, given the pleiotropic and complex nature of the hypoxic response and the large number of components involved in the HIF-1 system, it is important to develop very selective HIF-1 regulators for clinical applications. Systemic administration of small-molecule inhibitors of HIF-1 activity with anticancer effects that impairs both vascular and metabolic adaptations have been successfully used in a number of preclinical studies (138). However, no selective HIF-1α inhibitor has been clinically approved up to this date, mainly due to the nonspecific mode of action of these inhibitors affecting other pathways. The topical application of PHD inhibitors has been shown to improve wound healing in diabetic animals, representing a more immediate potential for clinical applications (139). Clinical trials of the PHD inhibitors FG-4592, GSK1278863, Molidustat, and Vadadustat, for anemia in chronic kidney disease are ongoing and appear to be encouraging (140). However, it is necessary to address the tissue-specific requirements of HIF-1 activity, temporal application of therapeutics, negative side effects of systemic applications, and potential non-specificity of therapeutic agents. Deciphering the role of non-coding RNAs, epigenetics, and different genetic factors underlying the complex networks of hypoxia/HIF-1α regulation may provide novel and more specific targets for future therapies.

## Author contributions

RC wrote the first draft of the manuscript. GP finalized and conceptualized the manuscript. All authors read and approved the submitted version.

### Conflict of interest statement

The authors declare that the research was conducted in the absence of any commercial or financial relationships that could be construed as a potential conflict of interest. The handling editor declared a shared affiliation, though no other collaboration, with the authors at time of review.
